# Fatigue Damage and Lifetime of SiC/SiC Ceramic-Matrix Composite under Cyclic Loading at Elevated Temperatures

**DOI:** 10.3390/ma10040371

**Published:** 2017-03-31

**Authors:** Longbiao Li

**Affiliations:** College of Civil Aviation, Nanjing University of Aeronautics and Astronautics, No. 29 Yudao St., Nanjing 210016, China; llb451@nuaa.edu.cn

**Keywords:** ceramic-matrix composites (CMCs), fatigue, damage evolution, life prediction, matrix cracking, interface debonding

## Abstract

In this paper, the fatigue damage and lifetime of 2D SiC/SiC ceramic-matrix composites (CMCs) under cyclic fatigue loading at 750, 1000, 1100, 1200 and 1300 °C in air and in steam atmosphere have been investigated. The damage evolution versus applied cycles of 2D SiC/SiC composites were analyzed using fatigue hysteresis dissipated energy, fatigue hysteresis modulus, fatigue peak strain and interface shear stress. The presence of steam accelerated the damage development inside of SiC/SiC composites, which increased the increasing rate of the fatigue hysteresis dissipated energy and the fatigue peak strain, and the decreasing rate of the fatigue hysteresis modulus and the interface shear stress. The fatigue life stress-cycle (S-N) curves and fatigue limit stresses of 2D SiC/SiC composites at different temperatures in air and in steam condition have been predicted. The fatigue limit stresses approach 67%, 28%, 39% 17% and 28% tensile strength at 750, 1000, 1100, 1200 and 1300 °C in air, and 49%, 10%, 9% and 19% tensile strength at 750, 1000, 1200 and 1300 °C in steam conditions, respectively.

## 1. Introduction

Ceramic materials possess a high strength and modulus at elevated temperatures. However, their use as structural components is severely limited because of their brittleness. Continuous fiber-reinforced ceramic-matrix composites, by incorporating fibers in ceramic matrices, however, not only exploit their attractive high-temperature strength but also reduce their propensity for catastrophic failure [1].

Many researchers have performed experimental and theoretical investigations on the cyclic fatigue behavior of fiber-reinforced ceramic-matrix composites (CMCs). Mall [2] investigated the effects of moisture on the cyclic fatigue behavior of a 2D SiC/SiC composite at 750 °C in air and in a humid environment. It was found that the presence of moisture decreased the fatigue life at a prescribed stress level relative to that without moisture. Michael [3] investigated the tension-tension fatigue behavior of 2D SiC/SiC composite at 1000 °C in air and in steam conditions. It was found that the presence of steam significantly degraded the fatigue performance, which accelerated the damage development and fatigue fracture due to oxidation embrittlement. Groner [4] investigated the cyclic fatigue behavior of 2D SiC/SiC composite with two geometries, unnotched and notched, at 1100 °C in air. It was found that the fatigue failure of the notched specimens was initiated adjacent to the hole and the failure of the unnotched specimens was initiated at the edge and inherent pores. Jacob [5] investigated the tension-tension fatigue behavior of 2D SiC/SiC composite at 1200 °C in air and in steam conditions. The microstructural investigation revealed pronounced oxidation on the fracture surface of specimens tested in steam. Ruggles-Wrenn and Lee [6] investigated the cyclic fatigue behavior of 2D SiC/SiC composite at 1300 °C in air and in steam conditions. It was found that the degradation of the fatigue performance at 1300 °C is mainly controlled by the fibers’ strength degradation. Ruggles-Wrenn and Lanser [7] investigated the tension-compression fatigue behavior of 2D woven Nextel™ 720/alumina composite at 1200 °C in air and in steam. The fatigue limit stress was achieved at 40% and 35% tensile strength in air and steam environments, respectively, when the maximum cycle number was defined as 100,000 applied cycles. The presence of steam noticeably degrades the tension–compression fatigue performance of the oxide/oxide composite. During cyclic loading, the damage evolution inside the composites should be monitored to predict the lifetime. Maillet et al. [8] investigated the damage evolution of 2D SiC/[Si-B-C] composite at temperatures of 450 °C and 500 °C using the acoustic emission (AE)-based approach during static fatigue loading. However, the AE-based approach used to damage monitoring is limited at elevated temperatures. Li [9,10] developed a hysteresis dissipated energy–based damage parameter for the damage evolution and life prediction of fiber-reinforced CMCs under cyclic fatigue loading at room and elevated temperatures. In the combustion process, substantial amounts of water vapor are produced from burning hydrocarbon fuels in air. Under equilibrium conditions, 5%–10% of the combustion gas is water vapor. The reactions of SiC fibers with water are therefore a concern. Yao et al. [11] investigated the effects of wet oxidation on the microstructural evolution, fracture mode and mechanical properties of Hi-Nicalon SiC fibers, and found that the water vapor enhances the oxidation rates. Park [12] investigated the effects of different oxidation conditions on the early-stage oxidation behavior of SiC fibers. The steam condition tests clearly yielded a lower O/Si ratio than the air oxidation test, which is likely related to the influence of volatilization on the concentration of the more oxygen-rich component (SiO_2_). Parthasarathy et al. [13] investigated the experimental grain growth and oxidation kinetics of SiC-based fibers and the accompanying strength degradation in argon, air, and moist air using a mechanistic model. The attendant loss in strength is shown in be captured by the model that proposes increases in the strength-limiting flaw size as being proportional to the grain growth.

The objective of this paper is to investigate the fatigue damage evolution and lifetime of 2D SiC/SiC composites at elevated temperatures. The damage development of 2D SiC/SiC composite was analyzed through the damage parameters of fatigue hysteresis dissipated energy, fatigue hysteresis modulus, fatigue peak strain and interface shear stress. The fatigue life stress-cycle (S-N) curves and fatigue limit stress of 2D SiC/SiC composites have been predicted.

## 2. Damage Parameters and Life Prediction Model

### 2.1. Damage Parameters

Genet et al. [14] investigated the crack network inside of 2D woven SiC/[Si-B-C] composite. It was found that matrix cracks exist in the yarns and the matrix outside of the yarns. Under cyclic fatigue loading, the matrix cracking modes in 2D woven CMCs can be divided into five different modes, including: [15].

(1) Mode 1: transverse cracking in the transverse tow, with debonding at the tow boundary;
(2) Mode 2: transverse cracking and matrix cracking with perfect fiber/matrix bonding and fracture of fibers occurs in the longitudinal tow;
(3) Mode 3: transverse cracking and matrix cracking with fiber/matrix debonding and sliding in the longitudinal tow;
(4) Mode 4: matrix cracking with perfect fiber/matrix bonding and fracture of fibers occurs in the longitudinal tow;
(5) Mode 5: matrix cracking and fiber/matrix interface debonding and sliding in the longitudinal tow.

The transverse yarns run perpendicular to the longitudinal yarns. Upon unloading and reloading, the relative frictional slip occurred in the fiber/matrix interface of the matrix cracking mode 3 and mode 5 [16].

For matrix cracking mode 3, the unloading strain $\varepsilon_{\mathrm{unload}}$ and reloading strain $\varepsilon_{\mathrm{reload}}$ are determined by Equations (1) and (2) [17].

(1) $$\varepsilon_{\mathrm{unload}}=\left\{ \begin{array}{l} \frac{\sigma }{V_{f}E_{f}}+4\frac{\tau_{i}}{E_{f}}\frac{y^{2}}{r_{f}l_{c}}-2\frac{\tau_{i}}{E_{f}}\frac{\left(2y-l_{d}\right)\left(2y-l_{c}+l_{d}\right)}{r_{f}l_{c}}-\left(\alpha_{c}-\alpha_{f}\right)\Delta Τ,l_{d}<\frac{l_{c}}{2}\\ \frac{\sigma }{V_{f}E_{f}}+4\frac{\tau_{i}}{E_{f}}\frac{y^{2}}{r_{f}l_{c}}-2\frac{\tau_{i}}{E_{f}}\frac{{\left(2y-l_{c}/2\right)}^{2}}{r_{f}l_{c}}-\left(\alpha_{c}-\alpha_{f}\right)\Delta Τ,l_{d}=\frac{l_{c}}{2} \end{array}\right.$$

(2) $$\varepsilon_{\mathrm{reload}}=\left\{ \begin{array}{l} \frac{\sigma }{V_{f\_\mathrm{axial}}E_{f}}-4\frac{\tau_{i}}{E_{f}}\frac{z^{2}}{r_{f}l_{c}}+\frac{4\tau_{i}}{E_{f}}\frac{{\left(y-2z\right)}^{2}}{r_{f}l_{c}}\\ \;+2\frac{\tau_{i}}{E_{f}}\frac{\left(l_{d}-2y+2z\right)\left(l_{d}+2y-2z-l_{c}\right)}{r_{f}l_{c}}-\left(\alpha_{c}-\alpha_{f}\right)\Delta Τ,l_{d}<\frac{l_{c}}{2}\\ \frac{\sigma }{V_{f}E_{f}}-4\frac{\tau_{i}}{E_{f}}\frac{z^{2}}{r_{f}l_{c}}+4\frac{\tau_{i}}{E_{f}}\frac{{\left(y-2z\right)}^{2}}{r_{f}l_{c}}-2\frac{\tau_{i}}{E_{f}}\frac{{\left(l_{c}/2-2y+2z\right)}^{2}}{r_{f}l_{c}}-\left(\alpha_{c}-\alpha_{f}\right)\Delta Τ,l_{d}=\frac{l_{c}}{2} \end{array}\right.$$

where *V*_f_ denotes the fiber volume content in the longitudinal direction; *E*_f_ denotes the fiber elastic modulus; *r*_f_ denotes the fiber radius; τ_i_ denotes the interface shear stress; *l*_c_ denotes the matrix crack spacing; *l*_d_ denotes the interface debonded length; *y* denotes the interface counter-slip length; *z* denotes the interface new-slip length; *α*_f_ and *α*_c_ denote the fiber and composite thermal expansion coefficient, respectively; ΔT denotes the temperature difference between the fabricated temperature T_0_ and the testing temperature T_1_ (ΔT = T_1_ − T_0_).

For matrix cracking mode 5, the unloading strain $\varepsilon_{\mathrm{unload}}$ and reloading strain $\varepsilon_{\mathrm{reload}}$ are determined by Equations (3) and (4) [17].

(3) $$\varepsilon_{\mathrm{unload}}=\left\{ \begin{array}{l} \frac{1}{V_{f}E_{f}}\left(\sigma -k\sigma_{\mathrm{to}}\right)+4\frac{\tau_{i}}{E_{f}}\frac{y^{2}}{r_{f}l_{c}}-2\frac{\tau_{i}}{E_{f}}\frac{\left(2y-l_{d}\right)\left(2y+l_{d}-l_{c}\right)}{r_{f}l_{c}}-\left(\alpha_{c}-\alpha_{f}\right)\Delta Τ,l_{d}<\frac{l_{c}}{2}\\ \frac{1}{V_{f}E_{f}}\left(\sigma -k\sigma_{\mathrm{to}}\right)+4\frac{\tau_{i}}{E_{f}}\frac{y^{2}}{r_{f}l_{c}}-2\frac{\tau_{i}}{E_{f}}\frac{{\left(2y-l_{c}/2\right)}^{2}}{r_{f}l_{c}}-\left(\alpha_{c}-\alpha_{f}\right)\Delta Τ,l_{d}=\frac{l_{c}}{2} \end{array}\right.$$

(4) $$\varepsilon_{\mathrm{reload}}=\left\{ \begin{array}{l} \frac{1}{V_{f}E_{f}}\left(\sigma -k\sigma_{\mathrm{to}}\right)-4\frac{\tau_{i}}{E_{f}}\frac{z^{2}}{r_{f}l_{c}}+\frac{4\tau_{i}}{E_{f}}\frac{{\left(y-2z\right)}^{2}}{r_{f}l_{c}}\\ \;+2\frac{\tau_{i}}{E_{f}}\frac{\left(l_{d}-2y+2z\right)\left(l_{d}+2y-2z-l_{c}\right)}{r_{f}l_{c}}-\left(\alpha_{c}-\alpha_{f}\right)\Delta Τ,l_{d}<\frac{l_{c}}{2}\\ \frac{1}{V_{f}E_{f}}\left(\sigma -k\sigma_{\mathrm{to}}\right)-4\frac{\tau_{i}}{E_{f}}\frac{z^{2}}{r_{f}l_{c}}+4\frac{\tau_{i}}{E_{f}}\frac{{\left(y-2z\right)}^{2}}{r_{f}l_{c}}\\ \;-2\frac{\tau_{i}}{E_{f}}\frac{{\left(l_{c}/2-2y+2z\right)}^{2}}{r_{f}l_{c}}-\left(\alpha_{c}-\alpha_{f}\right)\Delta Τ,l_{d}=\frac{l_{c}}{2} \end{array}\right.$$

Upon cyclic fatigue loading, the area associated with fatigue hysteresis loops is the energy lost during corresponding cycles, which is defined by Equation (5).

(5) $$U=\int_{\sigma_{\min}}^{\sigma_{\max}}\left[\varepsilon_{\mathrm{unload}}\left(\sigma \right)-\varepsilon_{\mathrm{reload}}\left(\sigma \right)\right]d\sigma$$

The fatigue hysteresis dissipated energy of matrix cracking modes 3 and 5 can be derived by inserting the corresponding unloading and reloading strains into Equation (5). The composite fatigue hysteresis dissipated energy is determined by Equation (6).

(6) $$U_{c}=\eta U_{3}+\left(1-\eta \right)U_{5}$$

where *U*_3_ and *U*_5_ denote the fatigue hysteresis dissipated energy of matrix cracking modes 3 and mode 5, respectively; and *η* is the damage parameter determined by the composite’s damage condition.

The fatigue hysteresis modulus *E* is defined by Equation (7).

(7) $$E=\frac{\sigma_{\max}-\sigma_{\min}}{\varepsilon_{c}\left(\sigma_{\max}\right)-\varepsilon_{c}\left(\sigma_{\min}\right)}$$

The increasing rate of peak strain ϕ is defined by Equation (8).

(8) $$\phi =\frac{\varepsilon_{\mathrm{peak}}\left(N_{\mathrm{final}}\right)-\varepsilon_{\mathrm{peak}}\left(N_{\mathrm{initial}}\right)}{N_{\mathrm{final}}-N_{\mathrm{initial}}}$$

where *ε*_peak_(*N*_final_) denotes the peak strain at the final applied cycle number of *N*_final_; and *ε*_peak_(*N*_initial_) denotes the peak strain at the initial applied cycle number of *N*_initial_.

The decreasing/increasing rate of the fatigue hysteresis dissipated energy is defined by Equation (9).

(9) $$\varphi =\left|\frac{U_{c}\left(N_{\mathrm{initial}}\right)-U_{c}\left(N_{\mathrm{final}}\right)}{N_{\mathrm{final}}-N_{\mathrm{initial}}}\right|$$

The degradation rate of the fatigue hysteresis modulus Φ is defined by Equation (10).

(10) $$\Phi =\frac{E\left(N_{\mathrm{initial}}\right)-E\left(N_{\mathrm{final}}\right)}{N_{\mathrm{final}}-N_{\mathrm{initial}}}$$

The degradation rate of the interface shear stress Ψ is defined by Equation (11).

(11) $$\Psi =\frac{\tau_{i}\left(N_{\mathrm{initial}}\right)-\tau_{i}\left(N_{\mathrm{final}}\right)}{N_{\mathrm{final}}-N_{\mathrm{initial}}}$$

### 2.2. Life Prediction Model

Under cyclic loading at elevated temperatures, fiber fracture occurred due to gradual interface wear and interface oxidation [18,19,20,21,22,23]. The global load-sharing assumption is used to determine the load carried by intact and fractured fibers [24].

(12) $$\frac{\sigma }{V_{f}}=\left[1-P_{f}\left(1+\frac{2l_{f}}{l_{c}}\right)\right]T+P_{r}\frac{2l_{f}}{l_{c}}\langle T_{b}\rangle$$

where *l*_f_ denotes the slip length over which the fiber stress would decay to zero if not interrupted by the far-field equilibrium stresses; and $\langle T_{b}\rangle$ denotes the average stress carried by broken fibers.

(13) $$P_{f}=\chi \left[\zeta P_{fa}+\left(1-\eta \right)P_{fb}\right]+P_{fc}+P_{fd}$$

(14) $$P_{r}=P_{fc}+P_{fd}$$

where *P_fa_*, *P_fb_*, *P_fc_* and *P_fd_* denote the fiber failure probability of oxidized fibers in the oxidation region, unoxidized fibers in the oxidation region, and fibers in the interface debonded region and interface bonded region, respectively; *ζ* denotes the oxidation fibers fraction in the oxidized region; and *χ* denotes the fraction of oxidation in the multiple matrix cracks.

(15) $$P_{fa}\left(T\right)=1-\exp\left\{-2\frac{L_{t}}{l_{0}}{\left[\frac{T}{\sigma_{0}\left(t\right)}\right]}^{m}\right\}$$

(16) $$P_{fb}\left(T\right)=1-\exp\left\{-2\frac{L_{t}}{l_{0}}{\left(\frac{T}{\sigma_{0}}\right)}^{m}\right\}$$

(17) $$P_{fc}\left(T\right)=1-\exp\left\{-\frac{r_{f}T^{m+1}}{l_{0}{\left(\sigma_{0}\left(N\right)\right)}^{m}\tau_{i}\left(N\right)\left(m+1\right)}\left[1-{\left(1-\frac{L_{d}\left(N\right)}{l_{f}\left(N\right)}\right)}^{m+1}\right]\right\}$$

(18) $$\begin{array}{l} P_{fd}\left(T\right)=1-\exp\{-\frac{2r_{f}T^{m}}{\rho l_{0}{\left(\sigma_{0}\left(N\right)\right)}^{m}\left(m+1\right)\left(1-\frac{\sigma_{\mathrm{fo}}}{T}-\frac{L_{d}\left(N\right)}{l_{s}\left(N\right)}\right)}\times [(1-\frac{L_{d}\left(N\right)}{l_{f}\left(N\right)}-\\ {\left(1-\frac{\sigma_{\mathrm{fo}}}{T}-\frac{L_{d}\left(N\right)}{l_{f}\left(N\right)}\right)\frac{\rho L_{d}\left(N\right)}{r_{f}})}^{m+1}-{\left(1-\frac{L_{d}\left(N\right)}{l_{f}\left(N\right)}-\left(1-\frac{\sigma_{\mathrm{fo}}}{T}-\frac{L_{d}\left(N\right)}{l_{f}\left(N\right)}\right)\frac{\rho L}{2r_{f}}\right)}^{m+1}]\} \end{array}$$

where *T* denotes the load carried by intact fibers; *r*_f_ denotes the fiber radius; *L*_d_ denotes the interface debonded length; *L* denotes the matrix crack spacing; *ρ* denotes the shear-lag model parameter; *σ*_fo_ denotes the fiber stress in the interface bonded region; *l*_f_ denotes the slip length over which the fiber stress would decay to zero if not interrupted by the far-field equilibrium stresses. The time-dependent fiber strength will be controlled by surface defects resulting from oxidation and is given by Equation (19) [21].

(19) $$\left\{ \begin{array}{l} \sigma_{0}\left(t\right)=\sigma_{0},t\leq \frac{1}{k}{\left(\frac{K_{IC}}{Y\sigma_{0}}\right)}^{4}\\ \sigma_{0}\left(t\right)=\frac{K_{IC}}{Y\sqrt[{4}]{kt}},t>\frac{1}{k}{\left(\frac{K_{IC}}{Y\sigma_{0}}\right)}^{4} \end{array}\right.$$

where *K*_IC_ denotes the critical stress intensity factor; *Y* is a geometric parameter; and *k* is the parabolic rate constant. Parthasarathy et al. [13] investigated the strength degradation of SiC fibers in air and in steam at elevated temperatures. The strength degradation of SiC fibers versus oxidation time curves at 750, 1000, 1100, 1200 and 1300 °C in air and steam conditions are illustrated in Figure 1, and predicted using Equation (19). The SiC fiber strength degradation with increasing time in SiC/SiC composite at elevated temperatures of 750, 1000, 1100, 1200 and 1300 °C in air and stream was predicted using Equation (19), and is shown in Figure 1.

With the increasing cycle number, the interface shear stress and fiber strength decrease due to the interface wear and interface oxidation [25]. The fiber failure probability in the interface oxidation region, interface debonded region and interface bonded region can be obtained by combining the interface wear model, interface oxidation model and fiber strength degradation model with Equations (12)–(14) [26]. The evolution of the fiber failure probability versus cycle number curves can be obtained. When the fiber broken fraction approaches the critical value, the composite fatigue fractures. The fatigue limit stress is calculated when the fracture applied cycles approach the maximum cycle number.

## 3. Experimental Comparisons

Under cyclic loading, the interface shear stress degrades with applied cycles. The interface shear stress degradation model developed by Evans has been used to determine the evolution of the interface shear stress [23].

(20) $$\left(\tau_{i}\left(N\right)-\tau_{s}\right)/\left(\tau_{0}-\tau_{s}\right)=\left(1+b_{0}\right){\left(1+b_{0}N^{j}\right)}^{-1}$$

where τ_0_ denotes the initial interface shear stress; τ_s_ denotes the steady-state interface shear stress; *b*_0_ is a coefficient; and *j* is an exponent which determines the rate at which interface shear stress drops with the number of cycles *N*.

### 3.1. Damage Evolution and Lifetime at 750 °C in Air

**Figure 2:**
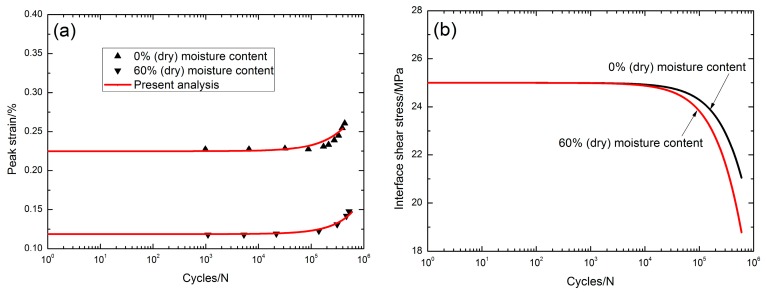

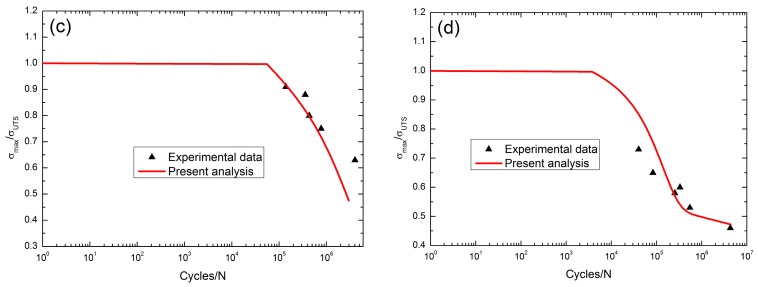
(**a**) The peak strain versus applied cycles with test environment of 0% moisture content condition and 60% moisture content condition; (**b**) the interface shear stress versus applied cycle with test environment of 0% moisture content condition and 60% moisture content condition; (**c**) the fatigue life S-N curve with test environment of 0% moisture content condition; and (**d**) with test environment of 60% moisture content condition of 2D SiC/SiC composite at 750 °C in air [2].

Mall [2] investigated the tension-tension fatigue behavior of 2D Syl-iBN/BN/SiC composite under two test environments, i.e., 0% and 60% moisture content conditions at 750 °C. The monotonic tensile strength was about 345 MPa. Under *σ*_max_ = 284 MPa and a 0% moisture content environment, the peak strain increased from 0.227% at the 984th applied cycle to 0.26% at the 434,323th applied cycle with the increasing rate of the peak strain of *ϕ* = 7.6 × 10^−10^/cycle, as shown in Figure 2a; the interface shear stress decreased from 25 MPa at the first applied cycle to 22 MPa at the 434,323th applied cycle with the degradation rate of the interface shear stress of Ψ = 6.9 × 10^−6^ MPa/cycle, as shown in Figure 2b. The interface shear stress degradation model parameters in Equation (20) are given in Table 1. Under *σ*_max_ = 190 MPa (55% *σ*_UTS_) and a 60% moisture content environment, the peak strain increased from 0.117% at the 1099th applied cycle to 0.147% at the 524,587th applied cycle, as shown in Figure 2a, with the increasing rate of the peak strain of *ϕ* = 5.7 × 10^−10^/cycle; the interface shear stress decreased slowly with applied cycles, i.e., from 25 MPa at the first applied cycle to 19.4 MPa at the 524,587th applied cycle, with the degradation rate of the interface shear stress of Ψ = 1.0 × 10^−5^ MPa/cycle, as shown in Figure 2b. The interface shear stress degradation model parameters in Equation (20) are given in Table 1. The oxidation and embrittlement of the fiber/matrix boron nitride (BN) interphase occurred in the presence of moisture to form the boria (B_2_O_3_) and reacted with SiC to form a borosilicate melt. When the maximum cycle number was defined to be 1,000,000 applied cycles, the fatigue limit decreased from 67% tensile strength under a 0% moisture environment to 49% tensile strength under a 60% moisture environment, as shown in Figure 2c,d.

### 3.2. Damage Evolution and Lifetime at 1000 °C

**Figure 3:**
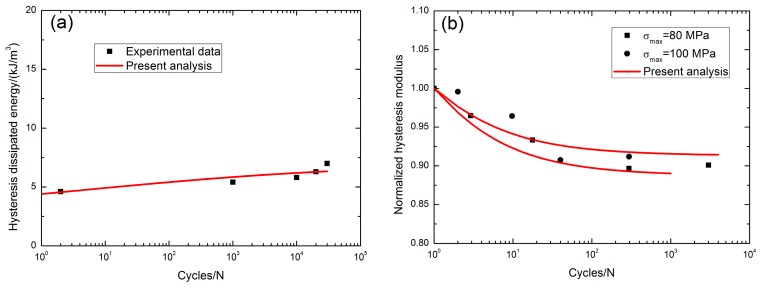

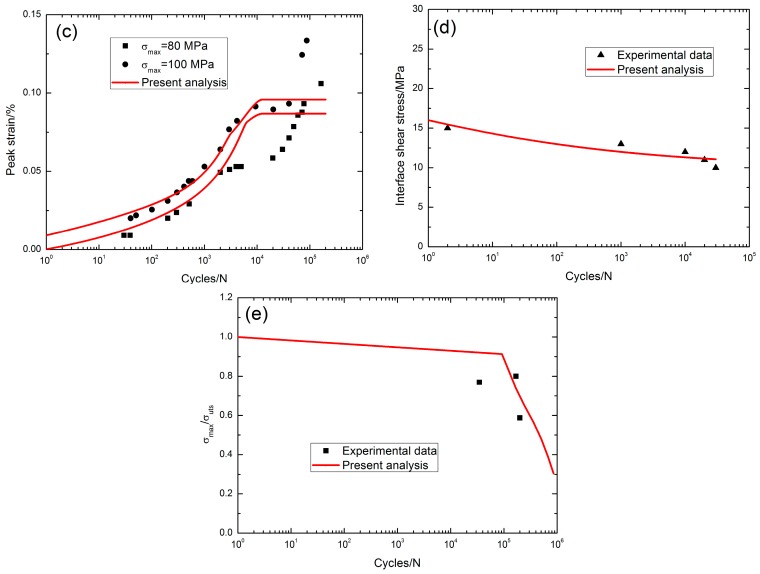
(**a**) The fatigue hysteresis dissipated energy versus applied cycles; (**b**) the normalized hysteresis modulus versus applied cycles; (**c**) the peak strain versus applied cycles; (**d**) the interface shear stress versus applied cycles; and (**e**) the fatigue life S-N curves of 2D SiC/SiC composite at 1000 °C in air [3].

Kanuf [3] investigated the tension-tension fatigue behavior of 2D CG Nicalon™/BN/SiC composite under two test environments, i.e., in air and in steam conditions at 1000 °C. The monotonic tensile strength was about 114 MPa. At 1000 °C in air, the fatigue hysteresis dissipated energy under *σ*_max_ = 80 MPa increases from 4.6 kJ/m^3^ at the second applied cycle to 7 kJ/m^3^ at the 30,000th applied cycle with the increasing rate of fatigue hysteresis dissipated energy of *φ* = 8 × 10^−5^ kJ·m^−3^/cycle, as shown in Figure 3a; the fatigue hysteresis modulus decreased from 1.0 at the first applied cycle to 0.9 at the 3022th applied cycle when *σ*_max_ = 80 MPa, and from 1.0 at the first applied cycle to 0.91 at the 295th applied cycle when *σ*_max_ = 100 MPa, as shown in Figure 3b. The fatigue peak strain increased from 0.009% at the 30th applied cycle to 0.106% at the 164122th applied cycle when *σ*_max_ = 80 MPa with the increasing rate of the fatigue peak strain of *ϕ* = 5.9 × 10^−9^/cycle, and from 0.02% at the 40th applied cycle to 0.133% at the 87,457th applied cycle when *σ*_max_ = 100 MPa with the increasing rate of the fatigue peak strain of *ϕ* = 1.3 × 10^−8^/cycle, as shown in Figure 3c; the interface shear stress decreased from 15 MPa at the second applied cycle to 10 MPa at the 30,000th applied cycle, with the interface shear stress degradation rate of Ψ = 1.6 × 10^−4^ MPa/cycle, as shown in Figure 3d, and the interface shear stress degradation model parameters in Equation (20) are listed in Table 1. The fatigue limit approached 28% tensile strength, as shown in Figure 3e.

**Figure 4:**
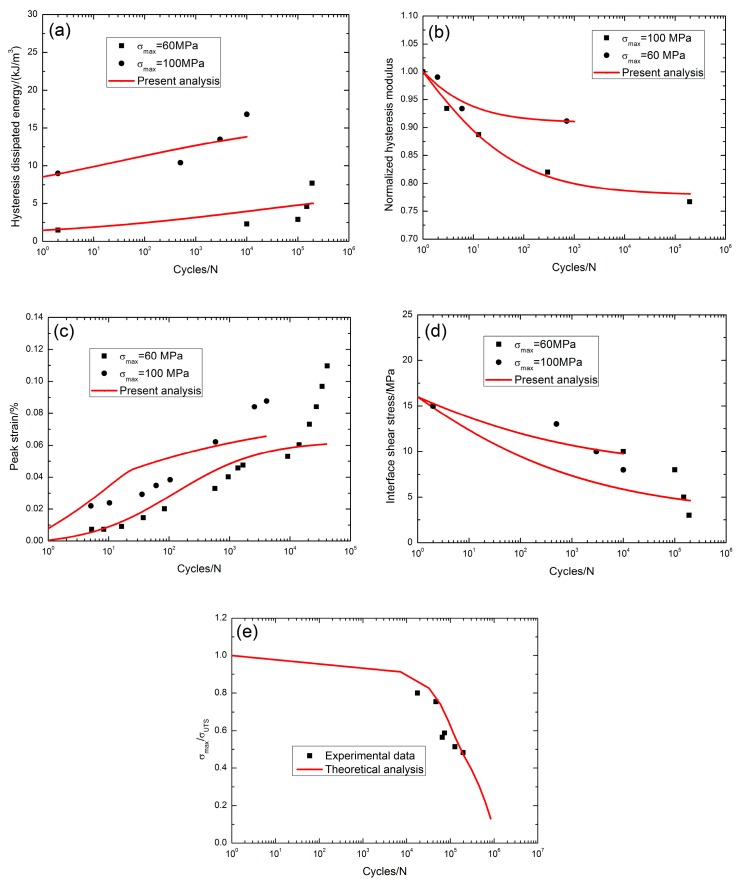
(**a**) The fatigue hysteresis dissipated energy versus applied cycles; (**b**) the normalized hysteresis modulus versus applied cycles; (**c**) the peak strain versus applied cycles; (**d**) the interface shear stress versus applied cycles; and (**e**) the fatigue life S-N curves of 2D SiC/SiC composite at 1000 °C in steam [3].

At 1000 °C in steam, the fatigue hysteresis dissipated energy increased from 1.5 kJ/m^3^ at the second applied cycle to 7.7 kJ/m^3^ at the 190,000th applied cycle with the increasing rate of the fatigue hysteresis dissipated energy of *φ* = 3.2 × 10^−5^ kJ·m^−3^/cycle when *σ*_max_ = 60 MPa, and from 9 kJ/m^3^ at the second applied cycle to 16.8 kJ/m^3^ at the 10,000th applied cycle with the increasing rate of the fatigue hysteresis dissipated energy of *φ* = 7.8 × 10^−4^ kJ·m^−3^/cycle when *σ*_max_ = 100 MPa, as shown in Figure 4a. The fatigue hysteresis modulus decreased from 1.0 at the first applied cycle to 0.76 at the 195,129th applied cycle when *σ*_max_ = 100 MPa, and from 1.0 at the first applied cycle to 0.91 at the 707th applied cycle when *σ*_max_ = 60 MPa, as shown in Figure 4b; the fatigue peak strain increased with applied cycles, i.e., from 0.007% at the fifth applied cycle to 0.109% at the 41,323th applied cycle when *σ*_max_ = 60 MPa with the increasing rate of the fatigue peak strain of *ϕ* = 2.4 × 10^−8^/cycle, and from 0.022% at the fifth applied cycle to 0.08% at the 4098th applied cycle when *σ*_max_ = 100 MPa with the increasing rate of the fatigue peak strain of *ϕ* = 1.4 × 10^−7^/cycle, as shown in Figure 4c. The interface shear stress decreased from 15 MPa at the second applied cycle to 3 MPa at the 190,000th applied cycle with the interface shear stress degradation rate of Ψ = 6.3 × 10^−5^ MPa/cycle when *σ*_max_ = 60 MPa, and from 15 MPa at the second applied cycle to 8 MPa at the 10,000th applied cycle with the interface shear stress degradation rate of Ψ = 7 × 10^−4^ MPa/cycle when *σ*_max_ = 100 MPa, as shown in Figure 4d, and the interface shear stress degradation model parameters in Equation (20) are listed in Table 1. The fatigue limit approached 10% tensile strength, as shown in Figure 4e. The presence of steam significantly degraded the fatigue performance of the SiC/SiC composite due to the oxidation of the BN interphase and SiC fibers. In the present analysis, the creep strain of the SiC fibers was not considered, leading to the difference between the theoretical analysis and experimental data, as shown in Figure 4c.

### 3.3. Damage Evolution and Lifetime at 1100 °C

**Figure 5:**
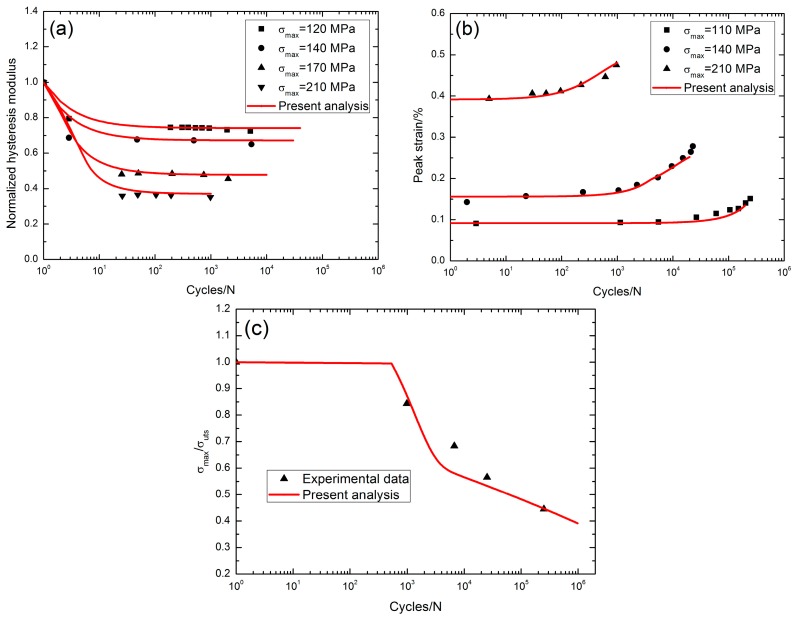
(**a**) The normalized fatigue hysteresis modulus versus applied cycles; (**b**) the peak strain versus applied cycles; and (**c**) the fatigue life S-N curves of 2D SiC/SiC composite at 1100 °C in air [4].

Groner [4] investigated the tension-tension fatigue behavior of 2D SiC/SiC composite at 1100 °C in air. The monotonic tensile strength was about 230 MPa. When *σ*_max_ = 120 MPa, the fatigue hysteresis modulus decreased from 1.0 at the first applied cycle to 0.72 at the 5102th applied cycle; when *σ*_max_ = 140 MPa, the fatigue hysteresis modulus decreased from 1.0 at the first applied cycle to 0.65 at the 5341th applied cycle; when *σ*_max_ = 170 MPa, the fatigue hysteresis modulus decreased from 1.0 at the first applied cycle to 0.455 at the 2042th applied cycle; and when *σ*_max_ = 210 MPa, the fatigue hysteresis modulus decreased from 1.0 at the first applied cycle to 0.35 at the 981th applied cycle, as shown in Figure 5a. The peak strain increased with applied cycles, i.e., when *σ*_max_ = 110 MPa, the peak strain increased from 0.091% at the third applied cycle to 0.151% at the 246,311th applied cycle with the increasing rate of the peak strain of *ϕ* = 2.4 × 10^−9^/cycle; when *σ*_max_ = 140 MPa, the peak strain increased from 0.142% at the second applied cycle to 0.278% at the 22,798th applied cycle with the increasing rate of the peak strain of *ϕ* = 5.9 × 10^−8^/cycle; and when *σ*_max_ = 210 MPa, the peak strain increased from 0.393% at the fifth applied cycle to 0.475% at the 965th applied cycle with the increasing rate of the peak strain of *ϕ* = 8.5 × 10^−7^/cycle, as shown in Figure 5b. The fatigue life S-N curve is shown in Figure 5c, and the fatigue limit approached 39% tensile strength at 1100 °C in air.

### 3.4. Damage Evolution and Lifetime at 1200 °C

**Figure 6:**
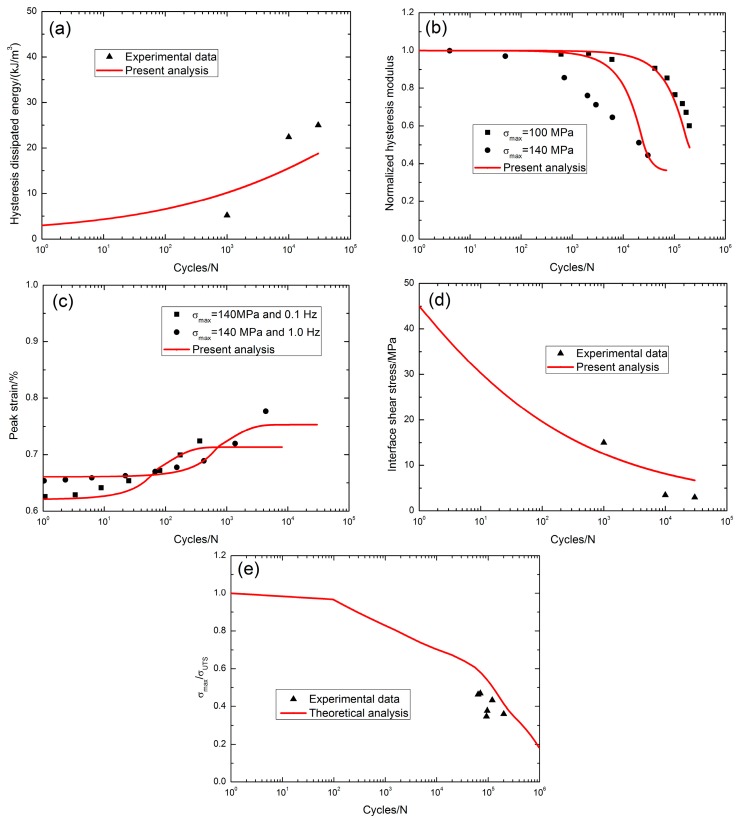
(**a**) The fatigue hysteresis dissipated energy versus applied cycles; (**b**) the normalized fatigue hysteresis modulus versus applied cycles; (**c**) the peak strain versus applied cycles; (**d**) the interface shear stress versus applied cycles; and (**e**) the fatigue life S-N curves of 2D SiC/SiC composite at 1200 °C in air [5].

Jacob [5] investigated the tension-tension fatigue behavior of 2D SiC/SiC composite under two test environments, i.e., in air and in steam conditions at 1200 °C. The monotonic tensile strength was about 306 MPa. At 1200 °C in air, when *σ*_max_ = 140 MPa (45.7%*σ*_UTS_), the fatigue hysteresis dissipated energy increased from 5.2 kJ/m^3^ at the 1000th applied cycle to 25 kJ/m^3^ at the 30,000th applied cycle with the increasing rate of the fatigue hysteresis dissipated energy of *φ* = 6.8 × 10^−4^ kJ·m^−3^/cycle, as shown in Figure 6a. The fatigue hysteresis modulus decreased with applied cycles, i.e., from 1.0 at the first applied cycle to 0.6 at the 196,841th applied cycle when *σ*_max_ = 100 MPa (32.6% *σ*_UTS_), and from 1.0 at the first applied cycle to 0.444 at the 30,509th applied cycle when *σ*_max_ = 140 MPa (45.7% *σ*_UTS_), as shown in Figure 6b. The fatigue peak strain increased with applied cycles, i.e., from 0.625% at the first applied cycle to 0.724% at the 362th applied cycle when *σ*_max_ = 140 MPa (45.7% *σ*_UTS_) and a loading frequency of 0.1 Hz with the increasing rate of the fatigue peak strain of *ϕ* = 2.7 × 10^−6^/cycle, and from 0.653% at the first applied cycle to 0.776% at the 4360th applied cycle when *σ*_max_ = 140 MPa (45.7% *σ*_UTS_) and a loading frequency of 1.0 Hz with the increasing rate of the fatigue peak strain of *ϕ* = 2.8 × 10^−7^/cycle, as shown in Figure 6c. The interface shear stress decreased with applied cycles, i.e., from 45 MPa at the 1000th applied cycle to 3 MPa at the 30,000th applied cycle with the interface shear stress degradation rate of Ψ = 1.4 × 10^−3^ MPa/cycle when *σ*_max_ = 140 MPa (45.7% *σ*_UTS_), as shown in Figure 6d; the interface shear stress degradation model parameters in Equation (20) are listed in Table 1. The fatigue life S-N curve is shown in Figure 6e, and the fatigue limit approached 17% tensile strength at 1200 °C in air.

**Figure 7:**
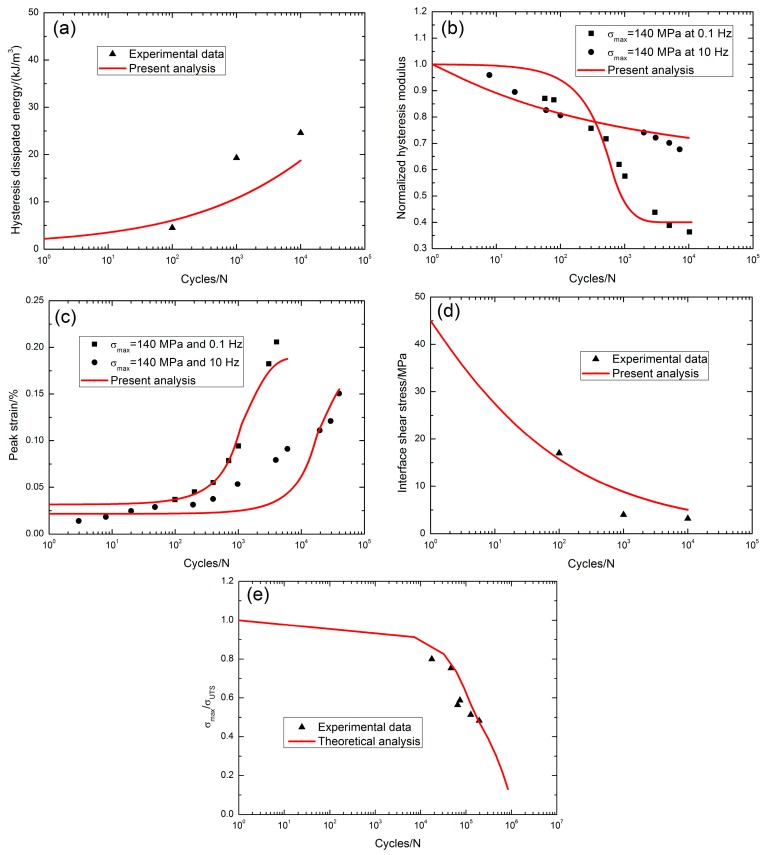
(**a**) The fatigue hysteresis dissipated energy versus applied cycles; (**b**) the normalized fatigue hysteresis modulus versus applied cycles; (**c**) the peak strain versus applied cycles; (**d**) the interface shear stress versus applied cycles; and (**e**) the fatigue life S-N curves of 2D SiC/SiC composite at 1200 °C in steam [5].

At 1200 °C in steam conditions, when *σ*_max_ = 140 MPa (45.7%*σ*_UTS_), the fatigue hysteresis dissipated energy increased from 4.5 kJ/m^3^ at the 100th applied cycle to 24.6 kJ/m^3^ at the 10,000th applied cycle with the increasing rate of the fatigue hysteresis dissipated energy of *φ* = 2.2 × 10^−3^ kJ·m^−3^/cycle, as shown in Figure 7a. The fatigue hysteresis modulus decreased with applied cycles, i.e., from 1.0 at the first applied cycle to 0.36 at the 10,236th applied cycle when *σ*_max_ = 140 MPa (45.7% *σ*_UTS_) with the loading frequency of 0.1 Hz, and from 1.0 at the first applied cycle to 0.67 at the 7208th applied cycle when *σ*_max_ = 140 MPa (45.7% *σ*_UTS_) with the loading frequency of 10 Hz, as shown in Figure 7b. The peak strain increased with applied cycles, i.e., from 0.036% at the 100th applied cycle to 0.205% at the 4043th applied cycle with the increasing rate of the peak strain of *ϕ* = 4.2 × 10^−7^/cycle when *σ*_max_ = 140 MPa (45.7%*σ*_UTS_) with the loading frequency of 0.1 Hz, and from 0.014% at the third applied cycle to 0.15% at the 39,737th applied cycle with the increasing rate of fatigue peak strain of *ϕ* = 3.4 × 10^−8^/cycle when *σ*_max_ = 140 MPa (45.7% *σ*_UTS_) with the loading frequency of 10 Hz, as shown in Figure 7c. The interface shear stress decreased with applied cycles, i.e., from 17 MPa at the 100th applied cycle to 3.2 MPa at the 10,000th applied cycle with the interface shear stress degradation rate of Ψ = 1.39 × 10^−3^ MPa/cycle, as shown in Figure 7d; the interface shear stress degradation model parameters in Equation (20) are listed in Table 1. The fatigue life S-N curve is shown in Figure 7e, and the fatigue limit approached 9% tensile strength at 1200 °C in steam atmosphere.

### 3.5. Damage Evolution and Lifetime at 1300 °C

**Figure 8:**
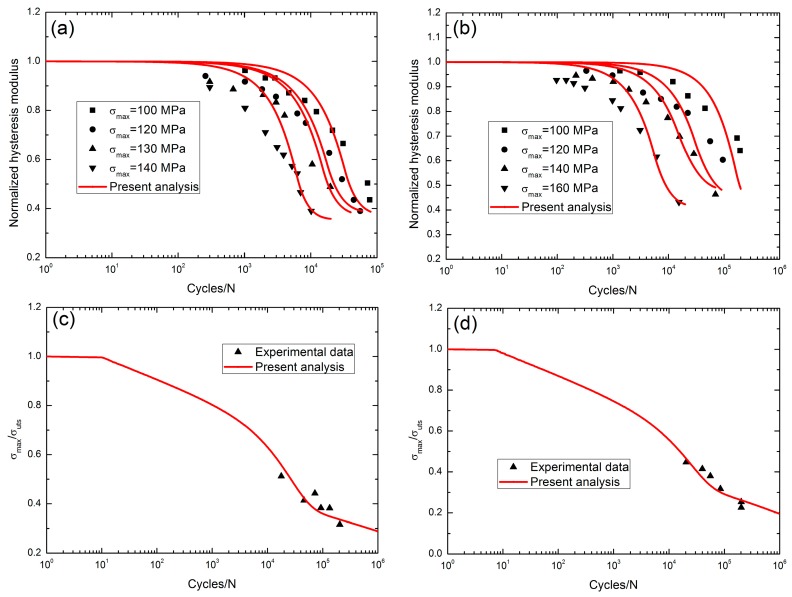
(**a**) The normalized fatigue hysteresis modulus versus applied cycles with the test environment in air; (**b**) the normalized fatigue hysteresis modulus versus applied cycles with test environment in steam; and (**c**) the fatigue life S-N curve with the test environment in air; and (**d**) the fatigue life S-N curve with the environment in steam of 2D SiC/SiC composite at 1300 °C [6].

Ruggles-Wrenn and Lee [6] investigated the tension-tension fatigue behavior of 2D Hi-Nicalon^TM^/SiC-B_4_C composite under two test environments, i.e., in air and in steam conditions, at 1300 °C. The monotonic tensile strength was about 311 MPa. At 1300 °C in air, the fatigue hysteresis modulus decreased with applied cycles, i.e., when *σ*_max_ = 100 MPa, the normalized fatigue hysteresis modulus decreased from 1.0 at the first applied cycle to 0.435 at the 77,639th applied cycle; when *σ*_max_ = 120 MPa, the normalized fatigue hysteresis modulus decreased from 1.0 at the first applied cycle to 0.389 at the 55,629th applied cycle; when *σ*_max_ = 130 MPa, the normalized fatigue hysteresis modulus decreased from 1.0 at the first applied cycle to 0.489 at the 19,719th applied cycle; and when *σ*_max_ = 140 MPa, the normalized fatigue hysteresis modulus decreased from 1.0 at the first applied cycle to 0.389 at the 10,124th applied cycle, as shown in Figure 8a. At 1300 °C in steam conditions, when *σ*_max_ = 100 MPa, the normalized fatigue hysteresis modulus decreased from 1.0 at the first applied cycle to 0.641 at the 194,352th applied cycle; when *σ*_max_ = 120 MPa, the normalized fatigue hysteresis modulus decreased from 1.0 at the first applied cycle to 0.603 at the 95,469th applied cycle; when *σ*_max_ = 130 MPa, the normalized fatigue hysteresis modulus decreased from 1.0 at the first applied cycle to 0.463 at the 70,087th applied cycle; and when *σ*_max_ = 140 MPa, the normalized fatigue hysteresis modulus decreased from 1.0 at the first applied cycle to 0.431 at the 15,414th applied cycle, as shown in Figure 8b. The fatigue life S-N curves at 1300 °C in air and in steam conditions are illustrated in Figure 8c,d, and the fatigue limit approached 28% tensile strength in air, and 19% tensile strength in steam conditions.

## 4. Discussion

At 750 °C in air, the fatigue strain increases with applied cycles, and the increasing rate of the peak strain increases with the peak stress and is affected by the test environment, i.e., *ϕ* = 7.6 × 10^−10^/cycle when *σ*_max_ = 284 MPa in a 0% moisture content, and *ϕ* = 5.7 × 10^−10^/cycle when *σ*_max_ = 190 MPa in a 60% moisture content; the fatigue limit decreases from 67% tensile strength under a 0% moisture environment to 49% tensile strength under a 60% moisture environment.

At 1000 °C, the degradation rate of the fatigue hysteresis modulus is higher in steam conditions than that in air, i.e., from 1.0 at the first applied cycle to 0.76 at the 195,129th applied cycle when *σ*_max_ = 100 MPa in steam, and from 1.0 at the first applied cycle to 0.91 at the 295th applied cycle when *σ*_max_ = 100 MPa in air; the increasing rate of the fatigue peak strain is higher in steam conditions than in air, i.e., *ϕ* = 1.4 × 10^−7^/cycle when *σ*_max_ = 100 MPa in steam conditions and *ϕ* = 1.3 × 10^−8^/cycle when *σ*_max_ = 100 MPa in air, and the fatigue limit stress in steam conditions is less than the fatigue limit stress in air, i.e., 10% tensile strength in steam versus 28% tensile strength in air.

At 1100 °C in air, the degradation rate of the fatigue hysteresis modulus increases with the fatigue peak stress, i.e., from 1.0 at the first applied cycle to 0.72 at the 5102th applied cycle when *σ*_max_ = 120 MPa and from 1.0 at the first applied cycle to 0.35 at the 981th applied cycle when *σ*_max_ = 210 MPa; the increasing rate of the fatigue peak strain increases with the fatigue peak strain, i.e., *ϕ* = 2.4 × 10^−9^/cycle when *σ*_max_ = 110 MPa versus *ϕ* = 8.5 × 10^−7^/cycle when *σ*_max_ = 210 MPa. The fatigue limit approaches 39% tensile strength at 1100 °C in air.

At 1200 °C, the increasing rate of the fatigue peak strain increases at a low loading frequency, i.e., when *σ*_max_ = 140 MPa in air, *ϕ* = 2.7 × 10^−6^/cycle with a loading frequency of 0.1 Hz versus *ϕ* = 2.8 × 10^−7^/cycle with a loading frequency of 1.0 Hz; when *σ*_max_ = 140 MPa in steam conditions, *ϕ* = 4.2 × 10^−7^/cycle with a loading frequency of 0.1 Hz versus *ϕ* = 3.4 × 10^−8^/cycle with a loading frequency of 10 Hz. The fatigue limit stress in steam conditions is less than the fatigue limit stress in air, i.e., 17% tensile strength at 1200 °C in air and 9% tensile strength at 1200 °C in steam atmosphere.

At 1300 °C, the degradation rate of the fatigue hysteresis modulus increases with the increasing fatigue peak stress, i.e., in air conditions, from 1.0 at the first applied cycle to 0.435 at the 77,639th applied cycle when *σ*_max_ = 100 MPa and from 1.0 at the first applied cycle to 0.389 at the 10,124th applied cycle when *σ*_max_ = 140 MPa; and in steam conditions, from 1.0 at the first applied cycle to 0.641 at the 194,352th applied cycle when *σ*_max_ = 100 MPa and from 1.0 at the first applied cycle to 0.431 at the 15,414th applied cycle when *σ*_max_ = 140 MPa. The fatigue limit stress in steam conditions is less than the fatigue limit stress in air, i.e., 28% tensile strength in air and 19% tensile strength in steam conditions.

## 5. Conclusions

The fatigue damage and lifetime of 2D SiC/SiC composites under cyclic fatigue loading at 750, 1000, 1100, 1200 and 1300 °C in air and in steam atmosphere have been investigated. The presence of steam accelerated the damage development inside of the SiC/SiC composites, which increased the increasing rate of the fatigue hysteresis dissipated energy and the fatigue peak strain, and the decreasing rate of the fatigue hysteresis modulus and the interface shear stress. The fatigue limit stresses approached 67%, 28%, 39% 17% and 28% tensile strength at 750, 1000, 1100, 1200 and 1300 °C in air, and 49%, 10%, 9% and 19% tensile strength at 750, 1000, 1200 and 1300 °C in steam conditions.

1. With the increase of the fatigue peak stress, the degradation rate of the fatigue hysteresis modulus and the interface shear stress increases, and the increasing rate of the fatigue peak strain and the fatigue hysteresis dissipated energy increases.
2. With the decrease of the loading frequency, the degradation rate of the fatigue hysteresis modulus and the interface shear stress increases, and the increasing rate of the fatigue peak strain and the fatigue hysteresis dissipated energy increases.

## Figures and Tables

**Figure 1:**
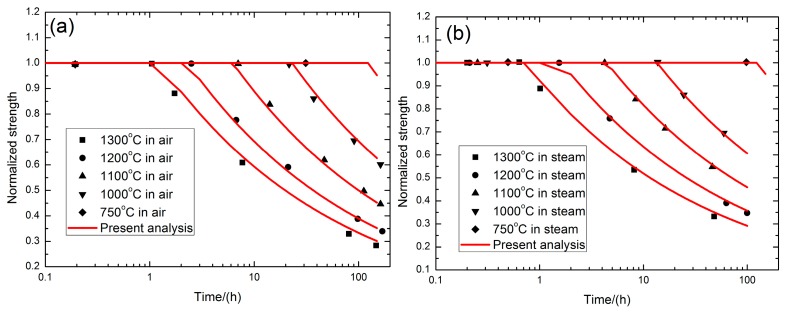
The strength degradation of SiC fibers (**a**) in air and (**b**) in steam conditions at elevated temperatures.

**Table 1:** The parameters of the interface shear stress degradation model for 2D SiC/SiC composite under different peak stresses, loading frequencies and test temperatures.

Temperatures	Environment	*σ*_max_/MPa	τ_0_/MPa	τ_s_/MPa	*b*_0_	*j*
750 °C	0% moisture content	284	25	1	1.0	5 × 10^−7^
	60% moisture content	190	25	1	1.0	3 × 10^−7^
1000 °C	Air	100	16	10	2.0	0.2
	Steam	100	16	3	2.0	0.3
1200 °C	Air	140	45	2	2.0	0.2
	Steam	140	45	1	2.0	0.3
